# Feelings about the timing of first sexual intercourse and health-related quality of life among adolescents

**DOI:** 10.1186/s12889-019-6728-y

**Published:** 2019-04-15

**Authors:** Manon Rouche, Katia Castetbon, Maud Dujeu, Estelle Méroc, Thérésa Lebacq, Camille Pedroni, Christelle Senterre, Isabelle Godin, Nathalie Moreau

**Affiliations:** 1 Service d’Information Promotion Education Santé (SIPES), School of Public Health, Université libre de Bruxelles (ULB), CP598, Route de Lennik 808, B-1070 Brussels, Belgium; 2 Research Centre in Epidemiology, Biostatistics and Clinical Research, School of Public Health, Université libre de Bruxelles (ULB), Brussels, Belgium; 3 Research Centre in Social Approaches to Health, School of Public Health, Université libre de Bruxelles (ULB), Brussels, Belgium

**Keywords:** Adolescents, Health-related quality of life, First sexual intercourse, HBSC, Belgium

## Abstract

**Background:**

Early sexual intercourse (SI) may have long-lasting negative impacts on health-related quality of life (HRQoL). So far, these impacts have been studied using age for defining early SI instead of feelings about its timing. The present study examined the association between feelings about the timing of first SI and current HRQoL.

**Methods:**

Data came from the 2014 cross-sectional Health Behaviour in School-aged Children (HBSC) study in French-speaking Belgium. Among participants aged 16–20 years who already had SI, 1778 were included in analyses. Univariate and multivariate logistic regressions were performed, including potential confounders.

**Results:**

One quarter of adolescents (26.4%) had poor HRQoL, 19.8% expressed a negative feeling about the timing of first SI and 19.6% did not think about it. Compared with adolescents who thought first SI happened at the right time or wished it had happened sooner, adolescents who had a negative feeling about the timing and those who did not think about it were more likely to have a poor HRQoL (cOR = 1.67 (1.28–2.17) and cOR = 1.37 (1.05–1.80), respectively). After adjustment, associations were no more significant (aOR = 1.22 (0.91–1.63) and aOR = 1.22 (0.91–1.64)). Sex disparity in expressing a negative feeling mostly explained the difference between crude and adjusted analyses.

**Conclusion:**

Further research is needed to better understand such a complex relationship. The high proportion of adolescents having poor HRQoL and negative feeling about the timing of first SI shows how important it is to find out effective prevention for both domains.

**Electronic supplementary material:**

The online version of this article (10.1186/s12889-019-6728-y) contains supplementary material, which is available to authorized users.

## Background

Health-related quality of life (HRQoL) is a multidimensional concept covering “physical, emotional, mental, social and behavioural components of well-being”, as perceived by children and adolescents [1]. Adolescence is a pivotal and critical period of the life span; it is marked by the onset of puberty and characterized by the development of identity and emotions [2]. Adolescents become physically and sexually mature and may need time to accept these changes. Besides those internal changes, social environment may also influence their behaviours. Adolescents may feel pressure to engage in risky behaviors, such as too early sexual intercourse (SI) that may have adverse physical and psychological consequences [2].

First SI, which involves mind and feelings [3], usually occurs in a loving relationship [4, 5]. Different studies have highlighted an association between early sexual initiation and psychological consequences [6–8]. Early sexual initiation may be associated with sexually transmitted infections (STIs), which could be explained by biological factors such as immature cervix [9, 10] and non-contraceptive use [4, 11], the latter also being associated with unintended pregnancy [4]. In turn, all these outcomes might have an impact on well-being [3, 12, 13], a component of the HRQoL. However, previous studies have addressed early sexual initiation based on the legal age of consent [6], i.e. the age above which someone is considered to be legally comptent to consent to a sexual act, or on associated risks [7, 8], rather than on individual perception or feelings.

Adolescents may express regrets about the timing of first SI, especially when it was triggered by pressure from friends or the partner [14, 15], or by sexual arousal [14]. Feeling that first SI happened too early or too late may have psychological consequences [14] including poor HRQoL.

Even if negative feelings are more frequent among adolescents who had first SI at a young age [16, 17], negative feelings are present at each age in proportions ranging from 29.1 to 55.0% according to the previous studies [15–19]. Furthermore, level of suitability of the relationship, level of preparedness, planning and willingness may be factors contributing to later regret [20].

The aim of this study was to describe the association between feelings about the timing of first SI and current HRQoL among 16–20 year old adolescents in French-speaking Belgium in 2014. Our hypothesis was that having a poor HRQoL was associated with feeling that first SI had happened too early.

## Methods

### Study design

The “Health Behaviour in School-aged Children” (HBSC) survey is a cross-sectional study conducted every 4 years in around 40 countries and regions under the aegis of the WHO Regional Office for Europe [21].

This study was carried out in the French-speaking part of Belgium among students from the fifth grade of elementary school (10 years of age) to the last grade of secondary school in 2014 (18–19 years in most situations). Questionnaires were self-administrated in the classroom under school-staff surveillance. Anonymity and data’s confidentiality were guaranteed [21]. Given the topics and the methods used for data collection, the protocol has not been submitted to an ethical committee but to the school authorities which allowed to carry out the survey.

### Sample

The survey was conducted on a random sample stratified proportionally to the school population distribution by province (*n* = 6) and school network (public and private) [21]. First, schools were randomly drawn; secondly, classes were randomly selected in each grade among schools which agreed to participate in the study. All pupils of selected classes were included in the sample [21].

**Fig. 1:**
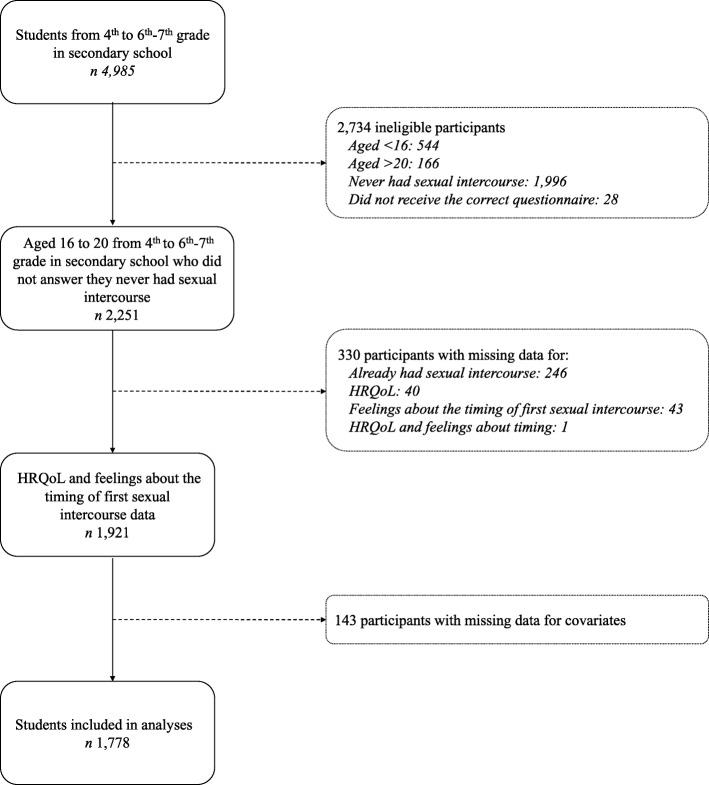
Inclusion diagram for students from 4th to 6th–7th grade in French-speaking Belgium, HBSC, 2014

In 2014, 394 secondary schools were invited to participate in the study. Among the schools that responded to the invitation (*n* = 193), 72 participated in the survey, which corresponds to a participation rate of 37.3% among respondents [21]. A total of 4985 questionnaires were collected for the students from the fourth to the sixth-seventh grades of secondary school. The number of students eligible in 2014 was 2251 (Fig. 1). Almost eight students out of ten fulfilled the eligible criteria and did not have missing data; thereby 1778 participants were included in analyses (Fig. 1).

### Measures

#### Feeling about the timing of first SI

This topic was explored with the question: “after your first sexual intercourse, did you think: (1) I wished it had happened sooner; (2) it happened at the right time; (3) I wished it had happened later; (4) I did not really want it to happen; (5) I did not think about it”. Due to small sample sizes and the closed meaning of categories, the grouping was as follows: negative feeling was represented by propositions (3) + (4), neutral feeling by (5) and reference category by (1) + (2). The intention of this question was to measure “full sexual intercourse” but other interpretation as a sexual practice could not be excluded.

#### HRQoL

The KIDSCREEN-10 was developed from the KIDSCREEN-27 and consists of a 10-item questionnaire [1]. Its reliability and validity as an independent instrument have been demonstrated [22]. The *T* value threshold of the HRQoL was set at 38, which corresponds to the 15th percentile of the distribution in the European reference population of teenagers aged from 12 to 18 years. All respondents with a *T* value lower than this threshold were considered as having a low HRQoL; this means that they “felt unhappy, incapable and dissatisfied with their family life, their relationships with peers and their lives in school” [1].

#### Perceived social support

The family and friend subscales are subjective social support instruments whose validity and reliability have been demonstrated [23]. The threshold values of the family and friend subscales were respectively set at 5.75 and 6.00, corresponding to the median of the score distribution in the whole sample. All the participants in the sample with a lower score than the threshold were considered as having a low social support [24].

#### Migration status

Adolescents born in Belgium with both parents born in Belgium were considered as natives. They were considered as second-generation migrants when they were born in Belgium while not both of their parents were born in Belgium. When neither the adolescents nor the two parents were born in Belgium, they were considered as adolescents of the first-generation. Adolescents who were born abroad but who had both parents born in Belgium were also considered as natives.

#### Affluence

The Family Affluence Scale (FAS) is a measure of family wealth used in adolescent surveys. The FAS is composed of six items and has been validated in Europe [25]. The FAS ranged from 0 to 13 and was divided into three categories - low, medium and high - corresponding respectively to scores 6 or below, between 7 and 9, 10 or higher.

In addition, age, sex, family structure, school orientation, daily smoking, drunkenness during life and cannabis experience were asked to the students.

### Statistical analyses

Statistical analyses were performed for students aged from 16 to 20 years from 4th grade to 6th–7th grade of secondary school who had had first SI and for whom all values in all variables of interest were available. Cramer’s V was used to assess the correlation between each independent variable.

Proportions of low HRQoL are reported for each category of variables. Comparisons of the proportions were carried out using the Pearson’s chi-squared test and Cochran-Armitage test for trend when appropriate. Univariate logistic regressions were performed between the dependent variable HRQoL and each variable of interest. Each explanatory variable was tested as confounding or effect modifier factor in bivariate analyses by the Mantel-Haenszel test of homogeneity with a threshold set at 0.05.

Using multivariate logistic regressions, association between feelings about the timing of first SI and a low HRQoL was assessed, accounting for all variables related to the HRQoL with a *P* value lower than 0.20 in univariate analyses to take into account potential confounding factors. A backward stepwise selection was used to determine the main model, which consisted in iteratively removing the least useful predictor that has a *P* value higher than 0.05. When removing a variable, confounding was assessed by checking the changes in the odds ratios (a tolerated variation was set at 10%) or drastic changes in the significance level for the remaining variables. Collinearity, fitting and specification of models were verified. Due to collinearity between drunkenness, daily smoking and cannabis experience, combining the three within a score was attempted but showed no significant association with HRQoL. Since drunkenness and cannabis experience were not individually associated with HRQoL, only daily smoking, that was associated with HRQoL in univariate analysis with a *p*-value < 0.20 (*p* = 0.18), was finally used in analyses.The threshold of statistical significance for all tests performed was set at 0.05.

Subsequently, to address the intermediate role of the context of first SI, variables related to that context were included in the main model by using a forward stepwise selection. Variables included dichotomized age at first SI (threshold set at 14 years old due to collinearity for other ages), contraceptive use at first SI (condom, pill, morning-after pill or another method) age difference with the partner and number of SI. Confounders and effect modifiers were assessed. Out of the 1778 participants of the main model, there was 4.8% missing data related to the context of first SI. Therefore, this subsequent multivariate model was performed on 1659 participants.

All analyses were performed using Stata/IC 12®.

## Results

### Participant characteristics

One quarter (26.4%) of adolescents had a low HRQoL, 19.8% reported a negative feeling about the timing of their first SI and 19.6% reported a neutral feeling (Table 1).

**Table 1:** Health behavior, and socio-demographic characteristics of the 16–20 year-old subjects. HBSC, French-speaking Belgium, 2014 (*n* = 1778)

Variables	n	%	Median (P25-P75)
Health-related quality of life^a,b^			
Low	470	26.4	
High	1308	73.6	
Feeling about timing of first sexual intercourse			
Wished it had happened sooner or it was right time	1078	60.6	
Wished it had happened later or did not really want it	352	19.8	
Did not think about it	348	19.6	
Age at interview^c^			
16 years old	289	16.2	
17 years old	562	31.6	
18 years old	442	24.9	
19–20 years old	485	27.3	
Sex			
Boys	846	47.6	
Girls	932	52.4	
Migration status^a^			
First-generation	190	10.7	
Second-generation	405	22.8	
Native	1183	66.5	
Family Affluence Scale^a^			
Low	393	22.1	8 (7–10)
Medium	897	50.4	
High	393	27.5	
Family structure			
Two biological parents	935	52.6	
Blended family	303	17.0	
Single-parent family	452	25.4	
Other	88	5.0	
Body image			
Too thin	37	2.1	
Thin	249	14.0	
Just right	732	41.2	
Fat	657	36.9	
Too fat	103	5.8	
School orientation			
Vocational	432	24.3	
Technical	643	36.2	
General	703	39.5	
Family support^a^			
Low	1010	56.8	5.50 (4.25–6.25)
High	768	43.2	
Friend support^a^			
Low	985	55.4	6.00 (5.00–6.75)
High	793	44.6	
Daily smoking			
Yes	374	21.0	
No	1404	79.0	

^a^For details, see Methods section

^b^Mean (SD) = 42.2 (7.2)

^c^Mean (SD) = 17.7 (1.2)

### Characteristics associated with low HRQoL

The timing of first SI was significantly associated with low HRQoL in univariate logistic regression (Table 2). Compared with adolescents in the reference category, adolescents who reported a negative feeling were more likely to have a low HRQoL (crude OR (cOR) = 1.67 (1.28–2.17)). This was also the case for those who reported a neutral feeling (cOR = 1.37 (1.05–1.80)). Sex, FAS, family structure, body image and perceived social support were significantly associated with a low HRQoL and therefore included in the initial multivariate model. Indeed, girls; adolescents with a low or medium FAS; from blended, single-parent or other family structures; declaring a “too thin”, “fat” or “too fat” body image; and having a low friend or family support were more likely to have a low HRQoL. Due to its *P* value lower than 0.20 in univariate analyses, smoking was also selected in multivariate analyses (Table 2).

**Table 2:** Univariate logistic regression for a low health-related quality of life^a^. HBSC, French-speaking Belgium, 2014 (*n* = 1778)

Variables	% low HQRoL	cOR (95% CI)^b^	*P* value
Feeling about timing of first sexual intercourse			
Wished it had happened sooner or it was right time	23.2	1	< 0.001
Wished it had happened later or did not really want it	33.5	1.67 (1.28–2.17)	
Did not think about it	29.3	1.37 (1.05–1.80)	
Age at interview			
16 years old	29.8	1	0.48
17 years old	24.7	0.78 (0.57–1.06)	
18 years old	26.2	0.84 (0.60–1.17)	
19–20 years old	26.6	0.85 (0.62–1.18)	
Sex			
Boys	18.9	1	< 0.001
Girls	33.2	2.14 (1.71–2.66)	
Migration status^b^			
First-generation	28.0	1.11 (0.80–1.54)	0.79
Second-generation	27.0	1.05 (0.82–1.35)	
Non-immigrant	26.0	1	
Family Affluence Scale^b^			
Low	34.9	2.24 (1.65–3.05)	< 0.001^c^
Medium	26.6	1.52 (1.16–1.99)	
High	19.3	1	
Family structure			
Two biological parents	22.1	1	< 0.001
Blended family	32.0	1.65 (1.24–2.21)	
Single-parent family	28.3	1.39 (1.08–1.80)	
Other	43.2	2.67 (1.71–4.19)	
Body image			
Too thin	43.2	3.01 (1.53–5.90)	< 0.001
Thin	24.5	1.28 (0.91–1.80)	
Just right	20.2	1	
Fat	30.1	1.70 (1.33–2.18)	
Too fat	45.6	3.31 (2.16–5.79)	
School orientation			
Vocational	23.6	1	0.31
Technical	27.2	1.21 (0.91–1.60)	
General	27.4	1.22 (0.93–1.61)	
Family support^b^			
Low	36.4	3.74 (2.93–4.78)	< 0.001
High	13.3	1	
Friend support^b^			
Low	33.7	2.41 (1.92–3.03)	< 0.001
High	17.4	1	
Daily smoking			
Yes	29.1	1.19 (0.92–1.53)	0.18
No	25.7	1	

^a^For details, see Methods section.

^b^Crude odds ratio and 95% confidence interval

^c^Cochran-Armitage test for trend

In the multivariate logistic regression, association between feelings about the timing of first SI and low HRQoL was no longer significant (Table 3). To find out what could explain such a change, all covariates were tested as potential confounding factors. Sex caused a variation of 14.4% from 1.42 (CI 95% 1.07–1.89) to 1.22 (CI 95% 0.91–1.63) of the odds of low HRQoL in the group of adolescents who reported a negative feeling. Nevertheless, sex had a slight impact in the group of adolescents who reported a neutral feeling. Family affluence scale, family structure, body image, perceived social support were still significantly associated with a low HRQoL in the multivariate logistic regressions. Indeed, girls; adolescents with a low FAS; from blended or other family structures; declaring a “too thin”, “fat” or “too fat” body image; and having a low friend or family support had higher odds of low HRQoL (Table 3). These covariates involved variations lower than 10%. No significant interaction was found and the sex stratified analysis did not provide any additional information.

**Table 3:** Multivariate^a^ logistic regression for low health-related quality of life^b^. HBSC, French-speaking Belgium, 2014 (n = 1778)

Variables	aOR (95% CI)^c^	*P* value
Feeling about timing of first sexual intercourse		
Wished it had happened sooner or it was right time	1	0.26
Wished it had happened later or did not really want it	1.22 (0.91–1.63)	
Did not think about it	1.22 (0.91–1.64)	
Sex		
Boys	1	< 0.001
Girls	2.20 (1.71–2.84)	
Family Affluence Scale ^b^		
Low	1.71 (1.22–2.41)	0.01
Medium	1.31 (0.98–1.76)	
High	1	
Family structure		
Two biological parents	1	0.04
Blended family	1.46 (1.07–1.99)	
Single-parent family	1.13 (0.85–1.50)	
Other	1.72 (1.05–2.81)	
Body image		
Too thin	3.33 (1.61–6.89)	< 0.001
Thin	1.36 (0.94–1.96)	
Just right	1	
Fat	1.40 (1.07–1.83)	
Too fat	2.12 (1.32–3,39)	
Family support^b^		
Low	3.22 (2.49–4.17)	< 0.001
High	1	
Friend support^b^		
Low	2.21 (1.73–3.39)	< 0.001
High	1	

^a^All variables were included in the final multivariate model

^b^For details, see Methods section

^c^Adjusted odds ratio and 95% confidence interval

### Potential intermediate role of the context of the first SI

The mean age at first SI was 15.6 (± 1.4) years, and 6.9% of adolescents had first SI before 14 years. Half (54.6%) had had their first SI with an older partner whereas 15.2% had it with a younger partner. Almost all used a contraceptive method (94.6%) and had subsequent SI (91.5%) (data not tabulated).

In the univariate logistic regression, only the number of SI was not associated with low HRQoL (*P* value > 0,20). Age difference with the partner (compared to same-age partner, younger partner: cOR = 0.98 (0.68–1.41), older partner: cOR = 1.44 (1.12–1.86)) and absence of contraceptive use (cOR = 1.73 (1.11–2.69)) were significantly associated with a low HRQoL (*P* value < 0,05) whereas age at first SI (cOR = 1.38 (0.91–2.09)) was not significantly associated with low HRQoL (*P* value =0,13).

In multivariate logistic regression, only the association between contraceptive use and low HRQoL was statistically significant (Additional File 1). Compared with the last main model, the contraceptive use changed the odds ratios of the feelings about the timing of first SI to 1.13 (95% CI 0.83–1.53, 7.4% of variation) for adolescents who reported a negative feeling, and to 1.21 (95% CI 0.89–1.66, 0.8% of variation) for adolescents who reported a neutral feeling.

## Discussion

Feelings about the timing of first SI was associated with HRQoL in univariate analyses: adolescents having a negative feeling of their first SI were more likely to have poor HRQoL than their peers wishing their first SI had happened sooner or was at the right time. However, this association disappeared in multivariate analyses, partly due to the differential distribution between sex and feelings (72.4% of girls reported negative feelings, compared with only 27.6% of boys). We also observed that HRQoL determinants like FAS and body image reduced the strength of the association between adolescents who reported a neutral feeling and poor HRQoL in multivariate analyses.

Referring to the definition of HRQoL [1, 26], HRQoL cannot be substituted for well-being. However, given the lack of literature on this topic, part of the discussion will be mainly oriented towards well-being.

The multidimensionality of well-being which contributes in HRQoL [1] and integrates “biological, psychological, social and spiritual dimensions” [27] could partly explain that feelings about the timing of first SI was not associated with HRQoL in multivariate analyses. It has been shown that the timing of first SI could have an impact on subsequent self-confidence as a competent sexual partner, which in turns had an impact on sexual well-being [28]. Sexual well-being can be measured by several indicators [29] derived from the WHO’s definition of sexual health that states it is “a state of physical, mental and social well-being in relation to sexuality” [30]. Thus, even if sexual well-being can be defined using the same dimensions as overall well-being, sexual well-being only represents one aspect of overall well-being and therefore of the HRQoL. Nevertheless, sexual well-being could not be evaluated in the context of our study. In addition, desire, willingness, and peer or partner pressure were not explored in our study, although they are major components of the context surrounding first SI [20, 29]. Another reason that could explain why association between feelings about the timing of first SI and HRQoL could not be demonstrated in multivariate analyses, lies in the wording used to express these feelings. It had been shown that regrets, regardless of the domain, are associated with poor subjective well-being [31]. In our questionnaire, we used a preference statement, expressing timing as “too early”, “too late” or “appropriate”, instead of a strong statement expressing regrets about the timing.

In view of the results obtained in the multivariate analysis, sex was identified as confounding factor. Sex disparity regarding the feelings about the timing of first SI can be explained by a greater degree of interpersonal sensitivity for girls than for boys. Girls tend to be more psychologically and emotionally involved in their interpersonal success about relationships [32] and hence may be more affected when they have relationship problems. Girls tend to idealize their first SI both at the setting and emotional level and hence to have negative feelings when it does not correspond to their expectations [33]. They also tend to feel more guilty about the first SI, especially when the first sexual relationship ends [33], leading to feeling of regret regarding the intercourse [13]. Girls are also more likely to engage in first SI because they want more intimacy and a stronger relationship [3, 34]. Meanwhile, boys are more likely to look for sexual pleasure [3] and to consider this act as an achievement [35]. In relation to their anatomy and to higher non-coital experience, males are more likely to have an orgasm and then a “physical gratification” during first SI [33]. On their side, girls can be physically affected by an unintended pregnancy and are at higher risk of contracting STI, due to a more favorable environment for penetration and growing of bacteria [36], that can induce psychological effects [13].

When subsenquently analyzing the circumstances in which the first SI occured, it appeared that adolescents who did not use contraception at first SI were more likely to have a poor HRQoL, even after adjusting for other covariates. This may be explained by the fact that they realized they took risks regarding STI or unplanned pregnancy [4], which may have an impact on well-being [12, 13]. Such risks and consequences on well-being could be different according to the contraceptive method. However, given that methods were highly correlated between each other (data not shown), using specific methods in the same modelling was not interpretable.

To our knowledge, this is the first study that investigates the association between feelings about the timing of first SI and HRQoL, hence comparison with other studies are limited. However, we observed in our study that the proportion of adolescents who reported a negative feeling was lower than the proportions documented in other studies [16–19]. In these studies, adolescents were asked to express what they thought about the timing of first SI at the time of the survey, whereas in our study we asked what they thought at the time of first SI. For instance, a cohort study presented by Sawyer and Smith in 1996 showed that proportion of students regretting their first SI were higher at a later time than immediately after it occurred [37].

Our study revealed that there was no significant association between feelings about the timing of first SI and HRQoL, partly due to the sex role. Indeed, in the multivariate analysis, the association became significant only when sex was removed from the model. This confounding situation is probably due to the fact that more girls than boys reported a low HRQoL on one hand, and a negative feeling on the other hand.

In our study, we evaluated the statistical association between feelings about the timing of first SI and HRQoL, instead of the causal relationship which would have rather required a prospective cohort design. Beyond to fulfill the descriptive purpose of the HBSC surveys [38], the cross-sectional design and a self-administrated questionnaire however made easier to include a large sample size and to reach a reliable precision in our estimations. The questionnaire was administrated during school hours, under the school-staff surveillance, including teachers. That condition could have induced biased results but they can be considered as limited. Indeed, instruction was given to ensure the confidentiality and anonymity of students’ answers by creating conditions of the type “examination” and by avoiding walking in the classroom.

Questions related to sexuality are considered as a sensitive topic and are subject to higher nonresponse rates and to higher measurement errors due to the social desirability [39]. If the social desirability bias was non-differential (i.e. adolescents underreported negative feelings in the same extent among those with a low HRQoL and among those with a high HRQoL), the association between feelings about the timing of first SI and HRQoL would tend toward the null.

Association between feelings about the timing of first SI and HRQoL might also have been underestimated because of selection bias. Indeed, the sex, migration status, friend support subscale, FAS and body image had a significant different distribution among the included participants and among the eligible who were not included due to missing data (data not shown). In addition, included participants had a higher HRQoL score than eligible participants not included in the analyses; low HRQoL was therefore probably underestimated and its association with feelings about the timing of first SI was then more likely to be underestimated.

Another limitation of this study was that the KIDSCREEN-10 was developed for the 8–18 years old children and adolescents whereas our study concerned adolescents from 16 to 20 years old. However, our findings are consistent with previous studies in that well-known determinants are associated with HRQoL in our study: for instance, adolescents in the group of low and medium FAS were more likely to have low HRQoL, compared with adolescents in the group of high FAS [22, 40]; girls were more likely than boys to have low HRQoL [1, 22].

## Conclusions

Further research is needed to confirm the lack of association due to sex between feelings about the timing of first SI and HRQoL, including for example sexual orientation, feeling of love, willingness or desire. These studies are necessary in order to effectively improve adolescent HRQoL and may help tailor the programs for health professionals, adolescents and their families. It is nevertheless already essential to find out adapted and effective preventive actions regarding negative feelings about the timing of first SI given the proportion observed. There is a need to support adolescents to develop control skills which might help to reduce these negative feelings. Since such feelings are more frequent among girls, focusing studies on girls without neglecting boys at risk, seems important.

## Additional File

Additional File 1: Multivariate^a^ logistic regression for low health-related quality of life^b^. HBSC, French-speaking Belgium, 2014 (*n* = 1659). (DOCX 18 kb)

## Abbreviations

95% CI: 95% Confidence interval
FAS: Family Affluence Scale
HBSC: Health Behaviour in School-aged Children
HRQoL: Health-Related Quality of Life
OR: Odds ratio
STI: Sexually transmitted infections
WHO: World Health Organization
