# Supplementary material for: Feelings about the timing of first sexual intercourse and health-related quality of life among adolescents
Source: BMC Public Health. 2019 Apr 15;19:408. doi: 10.1186/s12889-019-6728-y (PMC6466645; doi:10.1186/s12889-019-6728-y)
Supplement: Supplementary file 1 — Multivariatea logistic regression for low health-related quality of lifeb. HBSC, French-speaking Belgium, 2014 (n = 1659). (DOCX 18 kb) [file 12889_2019_6728_MOESM1_ESM.docx]

**Additional File 1.** Multivariate^a^ logistic regression for low health-related quality of life^b^

HBSC, French-speaking Belgium, 2014 (n=1,659)

| **Variables** | **aOR (95% CI)^b^** | ***P* value** |
| --- | --- | --- |
| **Feeling about timing of first intercourse**  Wished it had happened sooner or it was right time  Wished it had happened later or did not really want it  Did not think about it | 1  1.13 (0.83-1.53)  1.21 (0.89-1.66) | 0.43 |
| **Sex**  Boys  Girls | 1  2.33 (1.78-3.05) | <0.001 |
| **Family Affluence Scale** ^a^  Low  Medium  High | 1.71 (1.20-2.45)  1.27 (0.94-1.72)  1 | 0.01 |
| **Family structure**  Two parents  Recomposed family  Single-parent family  Other | 1  1.41 (1.02-1.95)  1.14 (0.84-1.53)  1.72 (1.05-2.81) | 0.09 |
| **Body image**  Too thin  Thin  Just right  Fat  Too fat | 3.29 (1.45-7.44)  1.40 (0.95-2.07)  1  1.37 (1.04-1.81)  2.01 (1.23-3,29) | <0.01 |
| **Family Subscale** ^c^  Low  High  **Friends Subscale** ^c^  Low  High | 3.34 (2.55-4.37)  1  2.29 (1.78-2.96)  1 | <0.01  <0.001 |
| **Contraceptive use**  Yes  No | 1  1.70 (1.04-2.80) | 0.04 |

^a^ All variables were included in the final multivariate model

^b^ For details, see Methods section

^c^ Adjusted odds ratio and its confidence interval
